# P-371. Few differences in persistence and virologic outcomes across age groups among CAB+RPV LA users: Findings from the OPERA Cohort

**DOI:** 10.1093/ofid/ofaf695.589

**Published:** 2026-01-11

**Authors:** Michael G Sension, Ricky K Hsu, Jennifer S Fusco, Laurence Brunet, Brooke Levis, Quateka Cochran, Gayathri Sridhar, Vani Vannappagari, Kimberley Brown, Jean A van Wyk, Michael B Wohlfeiler, Gregory P Fusco

**Affiliations:** can community health, Miami Beach, FL; AIDS Healthcare Foundation/ NYU School of Medicine, New York, NY; Epividian, Inc., Durham, North Carolina; Epividian, Inc., Durham, North Carolina; Epividian, Inc, Montreal, Quebec, Canada; Aids Healthcare Foundation, Miami Beach, Florida; ViiV Healthcare, Fairfax, Virginia; ViiV Healthcare, Fairfax, Virginia; ViiV Healthcare, Fairfax, Virginia; ViiV Healthcare, Brentford, UK, Brentford, England, United Kingdom; AIDS Healthcare Foundation, Miami, Florida; Epividian, Inc., Durham, North Carolina

## Abstract

**Background:**

Long-acting (LA) formulations of antiretrovirals may provide consistent therapeutic coverage with few drug interactions for older individuals living with HIV. Cabotegravir + rilpivirine (CAB+RPV) LA is the only complete LA antiretroviral therapy regimen approved for HIV-1 treatment in the United States. This study described persistence and virologic outcomes in individuals across different age groups receiving CAB+RPV LA injections in routine clinical care.

**Figure:**
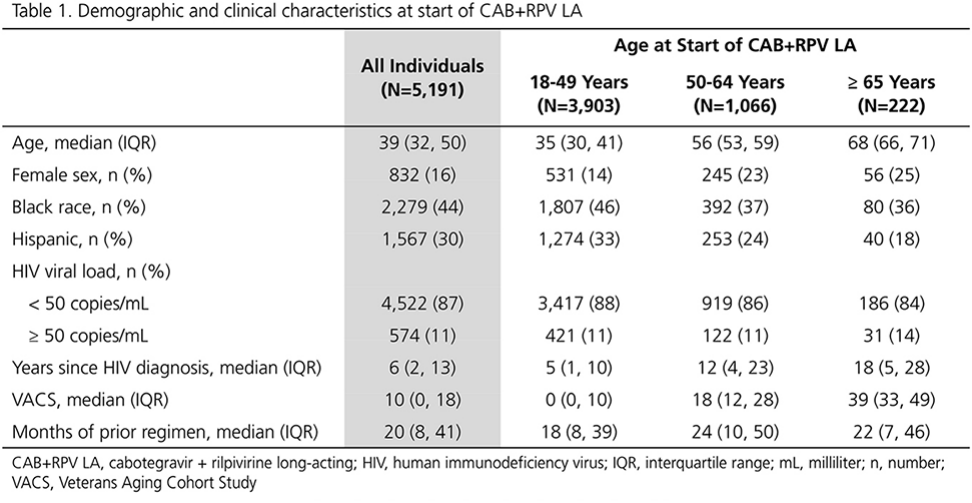

**Figure:**
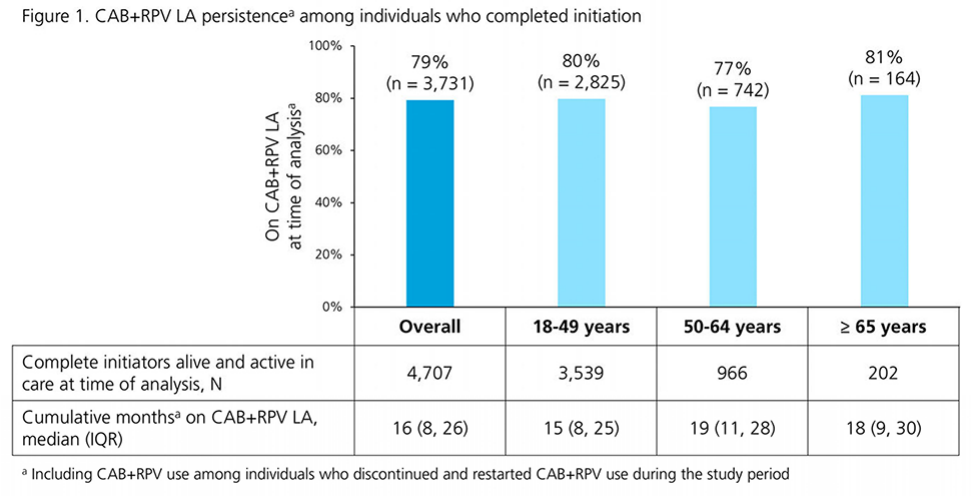

**Methods:**

Adults with HIV in the OPERA^®^ Cohort switching to CAB+RPV LA (21JAN2021-31DEC2024) regardless of viral load (VL) at initiation were followed through 29FEB2025. Among complete initiators (first 2 injections ≤ 67 days apart), we assessed persistence (on CAB+RPV LA at time of analysis) and suppression (VL < 50 copies/mL) at last measure. Outcomes were assessed overall and stratified by age (18-49, 50-64, ≥ 65 years) at initiation.

**Figure:**
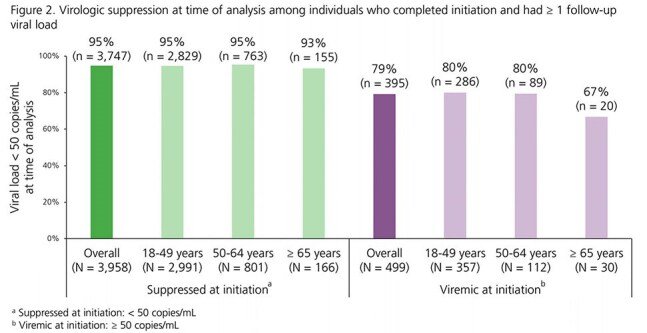

**Results:**

A total of 5191 individuals received CAB+RPV LA injections; 3903 (75%) were 18-49, 1066 (21%) were 50-64, and 222 (4%) were ≥ 65 years old. Compared to the youngest age group, individuals ≥ 50 years old were more likely to be female and less likely to be Black or Hispanic. They were also less likely to have VLs < 50 copies/mL, had HIV for longer, and had higher VACS scores at initiation (Table 1). Most (77-81%, depending on age group) complete initiators alive and in care at time of analysis were on CAB+RPV LA. The older age groups had slightly longer median months [interquartile range] durations on CAB+RPV LA (50-64: 19 [11, 28]; ≥ 65: 18 [9, 30]) than individuals aged 18-49 years (15 [8, 25]) (Fig 1). Among individuals with VL < 50 copies/mL at initiation, similarly high proportions were suppressed at last VL measure across age groups (18-49: 95%; 50-64: 95%; ≥ 65: 93%). Among individuals with VL ≥ 50 copies/mL at initiation, 67% of those aged ≥ 65 years were suppressed at last VL, compared to 80% of those aged 18-49 years or 50-64 years (Fig 2).

**Conclusion:**

In a large, diverse clinical population, over the first 4 years of CAB+RPV LA availability, adults with HIV were able to stay on regimen and remain suppressed across a wide range of ages. Among those viremic at initiation, most were suppressed to < 50 copies/mL at last measure with exception of a small group of ≥ 65 year-olds.

**Disclosures:**

Michael G. Sension, MD, Gilead: Grant/Research Support|Gilead: Honoraria|Viiv: Honoraria Ricky K. Hsu, MD, Gilead: Advisor/Consultant|Gilead: Grant/Research Support|Gilead: Honoraria|Merck: Honoraria|Serono: Advisor/Consultant|Serono: Honoraria|ViiV: Advisor/Consultant|ViiV: Grant/Research Support|ViiV: Honoraria Jennifer S. Fusco, BS, Gilead Sciences: Grant/Research Support|Merck & Co.: Grant/Research Support|Theratechnologies Inc: Grant/Research Support|ViiV Healthcare: Grant/Research Support Laurence Brunet, PhD, Gilead Sciences: Grant/Research Support|Merck & Co.: Grant/Research Support|Theratechnologies Inc.: Grant/Research Support|ViiV Healthcare: Grant/Research Support Brooke Levis, PhD, Gilead Sciences: Grant/Research Support|Merck & Co.: Grant/Research Support|TheraTechnologies: Grant/Research Support|ViiV Healthcare: Grant/Research Support Gayathri Sridhar, MBBS, MPH, PhD, GlaxoSmithKline: Stocks/Bonds (Public Company)|ViiV Healthcare: Full Time Employee Vani Vannappagari, MBBS, MPH, PhD, ViiV Healthcare: Full time Employee of ViiV Healthcare and owns GSK stock|ViiV Healthcare: Stocks/Bonds (Public Company) Kimberley Brown, PharmD, ViiV Healthcare: Employee|ViiV Healthcare: Stocks/Bonds (Public Company) Jean A. van Wyk, MBChB, MFPM, ViiV Healthcare: Employee|ViiV Healthcare: Stocks/Bonds (Public Company) Gregory P Fusco, MD, MPH, Gilead: Grant/Research Support|Merck: Grant/Research Support|Theratechnologies: Grant/Research Support|Viiv: Grant/Research Support

